# Utility of TyG-Based Indices for Predicting Insulin Resistance in Turkish Adults: Insights from the TEKHAP Study

**DOI:** 10.3390/jcm14248965

**Published:** 2025-12-18

**Authors:** Ayşe Kevser Demir, Şafak Şahin, Rıza Çıtıl, Osman Demir, Zeliha Cansel Özmen

**Affiliations:** 1 Department of Internal Medicine, Faculty of Medicine, Samsun University, 55090 Samsun, Türkiye; 2 Department of Internal Medicine, Faculty of Medicine, Tokat Gaziosmanpaşa University, 60250 Tokat, Türkiye; 3 Department of Public Health, Faculty of Medicine, Tokat Gaziosmanpaşa University, 60250 Tokat, Türkiye; 4 Department of Biostatistics, Faculty of Medicine, Tokat Gaziosmanpaşa University, 60250 Tokat, Türkiye; 5 Department of Biochemistry, Faculty of Medicine, Tokat Gaziosmanpaşa University, 60250 Tokat, Türkiye

**Keywords:** insulin resistance, triglyceride–glucose index, TyG-based indices, HOMA-IR, TG/HDL-C ratio, surrogate biomarker

## Abstract

**Background/Objectives:** Insulin resistance (IR) is a key feature of metabolic disorders and a major precursor of type 2 diabetes and cardiovascular disease. The triglyceride–glucose (TyG) index and TyG-based indices—including TyG–body mass index (TyG–BMI), TyG–waist circumference (TyG–WC), and TyG–waist-to-height ratio (TyG–WHtR), have been proposed as simple, cost-effective surrogate markers of IR. However, population-based data from Türkiye are limited. To evaluate the association between TyG-based indices, triglycerides/high-density lipoprotein cholesterol (TG/HDL-C) ratio, and IR defined by Homeostasis Model Assessment–IR (HOMA-IR) in adults from the TEKHAP study—a province-wide, population-based survey in Tokat, Türkiye. **Methods:** A total of 1854 adults (≥20 years) were included in the analysis. Physiologically implausible HOMA-IR outliers were identified using statistical criteria and excluded. IR was defined as HOMA-IR ≥ 2.46, previously validated in this population. TyG and its derivatives were calculated from fasting triglyceride, glucose, and anthropometric measurements. Group comparisons between IR and non-IR individuals, correlation analyses, receiver operating characteristic (ROC) curves, and multivariate logistic regression models were conducted to evaluate the diagnostic and independent associations of these surrogate markers with IR. **Results:** IR prevalence was 27.2%. Participants with IR had significantly higher triglycerides, fasting glucose, insulin, C-peptide, BMI, and waist circumference and lower HDL-C levels (all *p* < 0.001). All TyG-based indices were higher in the IR group and showed weak-to-moderate positive correlations with HOMA-IR. TyG–BMI showed the highest diagnostic accuracy in ROC curve analyses (AUC = 0.765); however, this association could not be interpreted as an independent predictive effect in adjusted models because of collinearity with BMI. In multivariable logistic regression, the TyG index demonstrated the strongest independent association with IR (OR = 4.14; 95% CI: 3.32–5.18; *p* < 0.001), while TyG–WC and the TG/HDL-C ratio also retained significant independent predictive value. **Conclusions:** The TyG index showed the strongest independent association with IR, while the TG/HDL-C ratio and TyG–WC also demonstrated significant independent predictive value. TyG-based indices may represent practical, low-cost surrogate markers for early metabolic risk stratification in community settings; however, their role in formal screening strategies requires external validation and calibration in independent populations.

## 1. Introduction

Insulin resistance (IR), defined as an impaired biological response to insulin stimulation in target tissues, leads to hyperglycemia and compensatory hyperinsulinemia due to increased insulin secretion from pancreatic beta cells. It predominantly affects the liver, skeletal muscle, and adipose tissue. IR is a common pathophysiological condition associated with various metabolic disorders, including obesity, prediabetes, type 2 diabetes mellitus (T2DM), and cardiovascular diseases [1]. The metabolic consequences of IR include endothelial dysfunction, elevated proinflammatory cytokine levels, and a prothrombotic tendency. Since IR is thought to develop approximately 10–15 years before the onset of T2DM, its early detection holds strategic importance for the prevention of chronic metabolic diseases [2].

The hyperinsulinemic–euglycemic clamp is considered the gold standard for assessing insulin sensitivity; however, it is not practical for routine use due to its complexity, cost, and laboratory requirements. The Homeostasis Model Assessment for IR (HOMA-IR) is a reliable and widely used alternative, yet its use in large-scale screenings is limited by the need for insulin measurements, which are costly and not routinely available in many laboratories. Therefore, there is growing interest in developing simple, inexpensive, and easily accessible biochemical markers that can serve as surrogate indicators of IR [3].

In this context, lipid profile parameters provide valuable indirect information about insulin sensitivity. Elevated triglyceride (TG) levels are considered a risk factor for IR, whereas high-density lipoprotein cholesterol (HDL-C) levels are protective. The triglyceride-to-HDL-C ratio (TG/HDL-C) has been reported to correlate more strongly with IR than either TG or HDL-C alone. This ratio reflects the atherogenic dyslipidemia pattern characterized by increased hepatic very low-density lipoprotein production and decreased HDL-C levels, which is considered an early indicator of both IR and endothelial dysfunction [4,5].

In recent years, the TyG index has gained considerable attention as a simple and reliable surrogate marker of IR. Multiple studies in diverse populations have demonstrated that the TyG index correlates strongly with HOMA-IR and predicts metabolic syndrome, type 2 diabetes, and cardiovascular outcomes [6,7,8]. Beyond the TyG index alone, TyG-based indices that incorporate anthropometric parameters—such as TyG–body mass index (TyG–BMI), TyG–waist circumference (TyG–WC), and TyG–waist-to-height ratio (TyG–WHtR)—have been proposed to enhance diagnostic performance for IR [8,9]. These composite indices integrate biochemical and anthropometric measures, potentially offering improved reflection of adiposity-related metabolic risk. Some studies have reported that TyG–BMI and TyG–WC may show stronger associations with IR and better discriminative capacity than the TyG index alone; however, their relative performance appears to vary across populations and methodological approaches [8,9].

Several recent large-scale studies and meta-analyses have strengthened the evidence supporting the diagnostic utility of the TyG index and TyG-based indices, highlighting their value not only in predicting IR but also in forecasting incident diabetes, metabolic syndrome, and other cardiometabolic outcomes [7,10,11]. These findings underscore the growing international relevance of TyG-based indices as practical and accessible surrogate markers of IR. However, in Türkiye, studies evaluating the association between TyG-based indices and IR in large, population-based cohorts remain scarce. The present study contributes to the national literature by examining these indices within a broad, population-representative adult sample.

The present study aimed to evaluate the associations between IR and surrogate markers, including the TG/HDL-C ratio and TyG-based indices (TyG–BMI, TyG–WC, and TyG–WHtR), using data from the “Prevalence of Chronic Diseases in Adults (TEKHAP)” study. Previously determined HOMA-IR cutoff values from the same population were applied, which is expected to enhance internal validity and allow population-based comparability of the results.

## 2. Methods

### 2.1. Study Design and Population

This study was designed as a cross-sectional, secondary analysis of data derived from the “Prevalence of Chronic Diseases in Adults (TEKHAP)” study, a population-based epidemiological study conducted in the Middle Black Sea region of Turkey. This dataset had been previously collected; therefore, the present study represents a retrospective cross-sectional secondary analysis. Data collection for the TEKHAP project was conducted between 2012 and 2013. The TEKHAP study was described in detail by Demir AK et al. [12] in their earlier publication titled “Prevalence of insulin resistance and identification of HOMA1-IR and HOMA2-IR indexes in the Middle Black Sea region of Turkey.” It employed a multistage proportional cluster sampling method that included both urban and rural areas of Tokat province and enrolled adults aged 20 to 84 years, ensuring a representative sample reflecting the provincial population pyramid by age and gender. This representative dataset provides a robust basis for further analyses.

**Figure 1:**
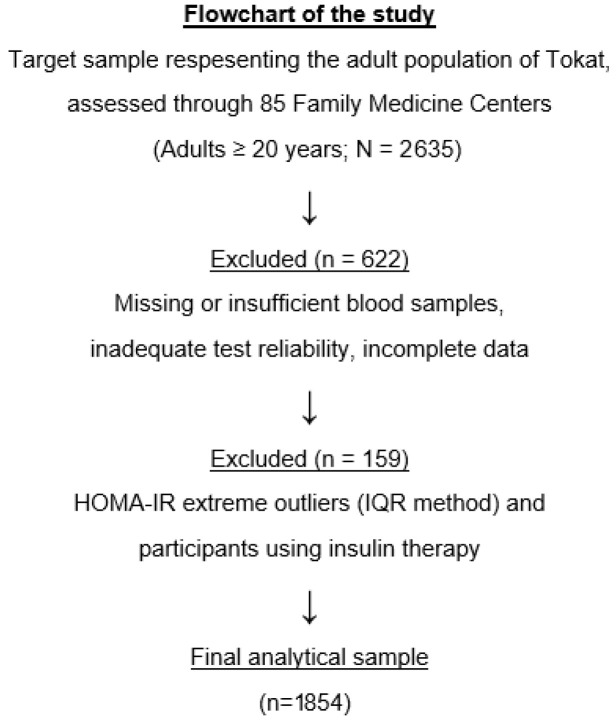
Flowchart of the study population selection process.

The present analysis was derived from the TEKHAP study, which initially recruited a representative sample of 2635 adults using a multistage, proportional cluster sampling strategy across 85 Family Medicine Center units in Tokat. Participants with missing or insufficient blood samples, impaired samples, inadequate test reliability, or incomplete biochemical or anthropometric data were excluded, resulting in 2013 eligible individuals. Additional exclusions were applied for physiologically implausible HOMA-IR values (identified using the IQR method) and for participants using insulin therapy to minimize treatment-related metabolic variability. The final analytical dataset consisted of 1854 participants with complete and physiologically plausible measurements. The number of participants excluded due to missing or inadequate data at each step is reported quantitatively in the flowchart (Figure 1).

### 2.2. Data Collection and Measurements

Blood samples were collected in the morning after an overnight fast of at least 10 h. All participants subsequently underwent standardized anthropometric and laboratory assessments. Body weight, height, and WC were measured using calibrated instruments, and BMI was calculated as weight (kg) divided by height squared (m^2^). The detailed protocol for fasting, blood sampling, biochemical analyses, and anthropometric assessments used in the TEKHAP study has been previously published [12] and is not repeated here to avoid redundancy.

Venous blood samples were collected to measure fasting plasma glucose, insulin, C-peptide, TG, and HDL-C. Biochemical parameters were analyzed using standardized enzymatic and immunoassay methods in an accredited central laboratory, in accordance with the protocols of the original TEKHAP study. All assays were performed under strict internal quality control procedures.

### 2.3. Definition of IR

IR was defined using HOMA-IR, calculated with the original HOMA model (HOMA1-IR), using the formula: (fasting glucose [mg/dL] × fasting insulin [µU/mL])/405 [13]. Although both HOMA1-IR and HOMA2-IR were calculated in our previous study [12] the term ‘HOMA-IR’ conventionally refers to the original HOMA1-IR model; therefore, HOMA-IR (HOMA1-IR) was used in the present analysis. The cut-off value of HOMA-IR ≥ 2.46, previously validated in the TEKHAP population, was applied.

The following formulas were applied for alternative surrogate markers [8,14,15]:

TG/HDL-C ratio = TG (mg/dL)/HDL-C (mg/dL)

TyG index = ln [TG (mg/dL) × Fasting glucose (mg/dL)/2]

TyG–BMI = TyG × BMI (kg/m^2^)

TyG–WC = TyG × Waist circumference (cm)

TyG–WHtR = TyG × WHtR (Waist circumference/height) (cm)

### 2.4. Statistical Analysis

All analyses were performed using IBM SPSS Statistics for Windows, version 25.0 (IBM Corp., Armonk, NY, USA). Data were expressed as mean ± standard deviation (SD) or percentage (%), as appropriate. Prior to analysis, the distribution of HOMA-IR values was examined, and extreme outliers were identified using the interquartile range (IQR) method. Participants with physiologically implausible HOMA-IR values were excluded to reduce undue influence on correlation and regression estimates.

Normality of continuous variables was assessed using histograms, Q–Q plots, and the Shapiro–Wilk test. Age, height, weight, BMI, waist circumference, WHR, and TyG-based indices demonstrated approximately symmetric distributions with mild right skew, whereas triglycerides, fasting glucose, insulin, C-peptide, HOMA-IR, and the TG/HDL-C ratio showed clear right-skewed distributions. In accordance with these findings, comparisons between IR and non–IR participants were performed using the Mann–Whitney U test for continuous variables and the chi-square test for categorical variables.

Correlations between HOMA-IR and TyG-based indices were evaluated using Pearson’s correlation coefficient (r). Residual diagnostic plots demonstrated approximately linear relationships without major deviations; therefore, Pearson correlation was considered appropriate. As an additional robustness check, Spearman’s rank correlation coefficients were also calculated and yielded a similar pattern of associations, confirming the stability of the findings.

The diagnostic performance of each index for predicting IR was assessed by Receiver Operating Characteristic (ROC) curve analysis, with calculation of area under the curve (AUC), optimal cut-off, sensitivity, and specificity values. Optimal cut-off values were determined using the Youden index (J = sensitivity + specificity − 1). For each index, 95% confidence intervals (CIs) were calculated for AUC, sensitivity, and specificity. Pairwise comparisons of AUCs were performed using DeLong’s test [16].

Binary logistic regression analyses were performed to determine the independent association between TyG-related indices and IR after adjustment for age, sex, and BMI. These covariates were selected because they are well-established determinants of insulin resistance and influence both TyG-based indices and HOMA-IR. Adjusting for these core confounders allows a more accurate evaluation of the independent contribution of each TyG-based index. A *p*-value < 0.05 was considered statistically significant.

### 2.5. Ethical Approval

The original TEKHAP study was approved by the Institutional Review Board of Gaziosmanpaşa University (approval code: 13-KAEK-024). For the present secondary analysis of the previously collected and anonymized TEKHAP dataset, additional ethical approval was obtained from the Research Ethics Committee of Tokat Gaziosmanpaşa University Faculty of Medicine (approval code: 25-MOBAEK-380) on 4 November 2025. The analysis complied with the principles of the Declaration of Helsinki.

## 3. Results

### 3.1. Baseline Characteristics of the Study Population

A total of 1854 participants were included in the final analysis. The mean (SD) age of the study population was 46.5 (±15.2) years, and 43.6% of the participants were male. Overall, 504 individuals (27.2%) were classified as having IR based on a HOMA-IR value ≥ 2.46. In sex-stratified analysis, the prevalence of IR was significantly higher in women than in men (29.7% vs. 23.9%, *p* = 0.007). Baseline demographic and anthropometric characteristics of the participants are presented in Table 1, while comparisons of anthropometric and biochemical parameters between individuals with and without IR are summarized in Table 2.

Individuals with IR had significantly higher levels of triglycerides, fasting glucose, body weight, waist circumference, insulin, and C-peptide compared to those without IR (all *p* < 0.001). Conversely, HDL-cholesterol levels were significantly lower in the insulin-resistant group (*p* < 0.001). In addition, height was significantly lower in individuals with IR (*p* < 0.001).

### 3.2. Comparison of TyG and TyG-Based Indices Between Groups

The TG/HDL-C ratio and all TyG-based indices—including the TyG index, TyG–BMI, TyG–WC, and TyG–WHtR—were significantly higher in the insulin-resistant group compared with the insulin-sensitive group (*p* < 0.001 for all) (Table 3). The largest mean differences were observed for TyG–BMI and TyG–WC, supporting their strong discriminatory potential of these composite indices.

### 3.3. Correlation Analyses

**Figure 2:**
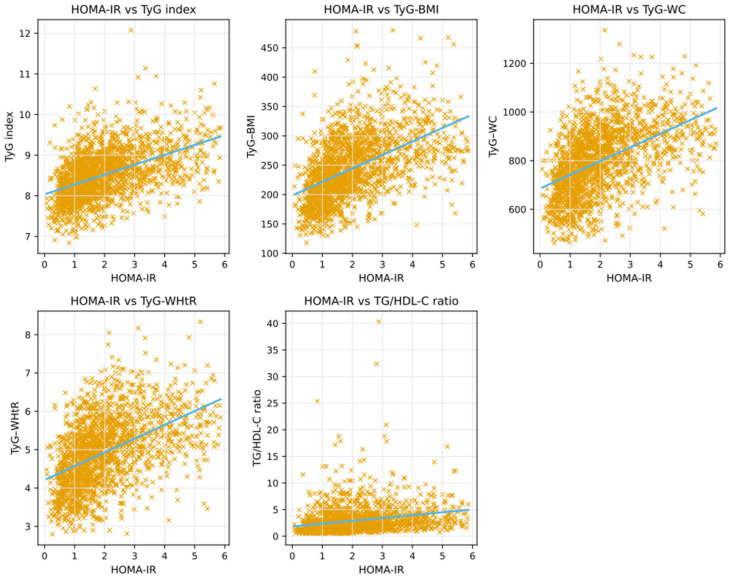
Scatter plots showing the correlations between HOMA-IR and TyG-based indices.

The correlations between HOMA-IR and TyG-based indices are summarized in Table 4, and their scatter plot representations are shown in Figure 2. All TyG-based indices demonstrated moderate, positive, and statistically significant correlations with HOMA-IR (all *p* < 0.001), whereas the TG/HDL-C ratio showed a weaker yet still significant positive correlation. Among all indices, TyG–BMI exhibited the strongest correlation with HOMA-IR (r = 0.515), followed by TyG–WHtR and TyG–WC, while the TG/HDL-C ratio demonstrated the weakest association.

### 3.4. Receiver Operating Characteristic (ROC) Curve Analyses

**Figure 3:**
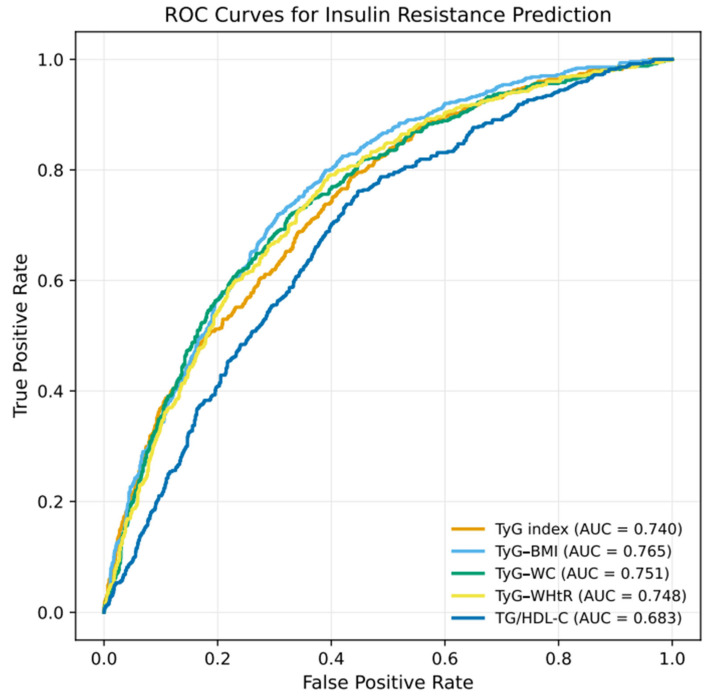
ROC Curves for TyG-Based Indices and TG/HDL-C Ratio in Predicting IR.

The diagnostic performance of the TyG-based indices for predicting IR is presented in Table 5. All indices demonstrated statistically significant discriminatory ability, with AUC values exceeding 0.68. The corresponding ROC curves are presented in Figure 3, illustrating the comparative predictive performance of each index. Among the evaluated indices, TyG–BMI showed the highest discriminative performance (AUC = 0.765), followed by TyG–WC (AUC = 0.751), TyG–WHtR (AUC = 0.748), and the TyG index (AUC = 0.740). In contrast, the TG/HDL-C ratio exhibited lower discriminative ability (AUC = 0.683) compared with the TyG-based indices.

### 3.5. Logistic Regression Analyses

As shown in Table 6, univariate logistic regression analysis demonstrated that all TyG-based indices and the TG/HDL-C ratio were significantly associated with the presence of IR (all *p* < 0.001). The strongest associations were observed for the TyG index (OR = 4.68, 95% CI: 3.81–5.75) and TyG–WHtR (OR = 2.86, 95% CI: 2.49–3.30). After multivariable adjustment for age, sex, and BMI (Table 7), the TyG index, TyG–WC, and the TG/HDL-C ratio remained independent predictors of IR. In contrast, other TyG-based indices, including TyG–BMI and TyG–WHtR, lost statistical significance in the adjusted models, likely due to collinearity with BMI and waist circumference. Neither age nor sex was independently associated with IR in the fully adjusted model (both *p* > 0.05).

## 4. Discussion

This study analyzed data from the TEKHAP cohort to explore the association of TyG-related indices and the TG/HDL-C ratio with IR defined by HOMA-IR. It represents one of the few population-based studies in Türkiye addressing these markers in a representative adult population.

The TEKHAP study is a province-wide, community-based study conducted using a multistage, stratified cluster sampling method encompassing both urban and rural areas. This design enhances the external validity of the findings by providing a more realistic representation of metabolic risk at the population level. In Türkiye, most previous studies [17,18] on this subject have been hospital-based or limited by small sample sizes. For example, Kırtıl et al. [19] reported a significant but weak correlation between the TyG index and HOMA-IR (r = 0.21) in a cohort of adult individuals. In contrast, the broader and standardized structure of the TEKHAP study sample allows for improved generalizability and greater analytical strength in the evaluation of TyG-based indices.

Our results demonstrated that all TyG-based indices, as well as the TG/HDL-C ratio, were significantly associated with IR and showed good discriminatory performance. Although TyG–BMI and TyG–WC exhibited the highest diagnostic accuracy in ROC analyses, multivariable models revealed that only the TyG index, TyG–WC, and the TG/HDL-C ratio remained independent predictors of IR, suggesting that the apparent superiority of TyG–BMI in unadjusted analyses may be attenuated by its strong correlation with BMI. These findings align with previous international studies [20,21] supporting the TyG index and TyG-based indices as practical surrogate markers of IR. Guerrero-Romero et al. [22] originally introduced the TyG index by comparing it with the euglycemic–hyperinsulinemic clamp technique, reporting a strong correlation between the two methods. Subsequently, Song et al. [9], in a cross-sectional analysis based on KNHANES data, demonstrated that TyG-based indices incorporating anthropometric parameters improved the predictive performance for IR, particularly among younger adults. Further studies have consistently confirmed that composite indices combining the TyG index with anthropometric measures—such as TyG–BMI and TyG–WC—enhance the diagnostic accuracy for identifying individuals with IR. Our findings are in line with these observations, demonstrating that TyG–BMI and TyG–WC exhibited the highest diagnostic performance, while the TyG index and TyG–WC remained independent predictors in multivariate models. Overall, these results support the relevance of the TyG index in population-based analyses, while the interpretation of composite TyG-based indices should account for their underlying anthropometric components.

Similarly, in a study [23] including 13,908 participants from three different cohorts, the TG/HDL-C ratio was reported to be strongly associated with metabolic syndrome (OR ranging from 3.5 to 6.0), IR defined by HOMA-IR (*p* < 0.0001), and the severity of carotid atherosclerosis (*p* = 0.0001). These findings support the utility of the TG/HDL-C ratio as a practical biomarker for identifying IR and metabolic risk. Likewise, Abdul Ghani et al. [5] demonstrated that in healthy Iraqi adults, the TG/HDL-C ratio served as a strong predictor of IR, providing 83% sensitivity and 81% specificity at a cut-off value of 3.1. In our TEKHAP cohort, the TG/HDL-C ratio showed a weak but statistically significant positive correlation with HOMA-IR (r ≈ 0.25, *p* < 0.001). Our findings suggest weaker correlations, likely reflecting population differences and the relatively lower discriminatory capacity observed in our dataset. In line with this, a recent systematic review by Baneu et al. [4], which included 32 studies encompassing nearly 50,000 participants, confirmed a significant positive association between the TG/HDL-C ratio and IR across diverse populations. However, the authors emphasized that the diagnostic performance and optimal cut-off values of this ratio vary according to ethnicity, sex, and age, and that the TG/HDL-C ratio alone may have limited discriminatory power. They suggested that combining this marker with biochemical or anthropometric parameters—such as TyG-based indices—could further improve the accuracy of IR prediction.

In a large Korean cohort, Lim et al. [8] reported adjusted odds ratios (ORs) for predicting IR of 7.60 for TyG, 12.82 for TyG–BMI, 16.29 for TyG–WC, and 14.86 for TyG–WHtR when comparing the highest and lowest quartiles. These findings indicate a strong association between TyG-based indices and IR, highlighting TyG–WC as the most powerful predictor among the evaluated indices. In our TEKHAP cohort, TyG–BMI and TyG–WC similarly demonstrated the highest diagnostic accuracy in ROC analyses, indicating meaningful discriminative ability for identifying IR. However, in multivariable models, the TyG index emerged as the strongest independent predictor, suggesting that its predictive value is more robust after accounting for anthropometric and demographic factors.

These results suggest that combining biochemical parameters (triglycerides and fasting glucose) with anthropometric measurements (such as waist circumference and BMI) may enhance the characterization of metabolic risk compared to biochemical markers alone. Importantly, discriminative ability assessed by ROC analyses reflects how well an index separates individuals with and without IR, whereas independent predictive value in multivariable models indicates whether an index contributes information beyond established confounders. Accordingly, although composite TyG-based indices may show higher AUCs, strong collinearity with anthropometric measures limits their interpretation as independent predictors.

From a practical standpoint, TyG-based indices are low-cost, non-invasive, and easily applicable, relying on routine fasting glucose and lipid measurements. These characteristics support their use for early population-level risk stratification, particularly in settings where insulin assays are not routinely available; however, their application in formal screening should be considered exploratory until external validation and calibration are established.

### 4.1. Strengths and Limitations

The major strengths of this study include its large, province-wide, population-based design; the use of standardized measurement protocols; and the application of a locally validated HOMA1-IR threshold derived from the same cohort, which enhances internal validity. To our knowledge, this is one of the first studies in Türkiye to evaluate the TyG index and TyG-based indices in a broadly representative adult population.

However, several limitations should be acknowledged. First, the cross-sectional design precludes any causal inference. Second, although biochemical measurements were obtained using standardized laboratory methods, single fasting values may not fully capture intra-individual metabolic variability. Third, lifestyle-related factors such as dietary habits and physical activity were not assessed, which may have contributed to variability in insulin sensitivity. Additionally, using a single HOMA1-IR cut-off may result in some degree of misclassification, as insulin resistance is a continuous trait, and residual confounding from unmeasured lifestyle factors cannot be excluded.

Because the study population reflects a community-based sample, individuals with diabetes were not excluded. Although this enhances representativeness, the presence of diabetes could influence the distribution of metabolic markers. This potential impact was minimized by excluding physiologically implausible HOMA-IR outliers using the interquartile range (IQR) method and removing participants using insulin therapy to reduce treatment-related confounding. Furthermore, although age, sex, and BMI were included as core confounders in multivariable models, additional metabolic covariates were not incorporated to avoid multicollinearity; this omission is acknowledged as a limitation.

Additionally, several methodological limitations should be noted.

- The diagnostic performance of TyG-based indices was not externally validated in an independent dataset, which limits generalizability beyond the TEKHAP population.
- Although ROC analyses were performed, calibration measures—such as calibration plots or Brier scores—were not evaluated, preventing a full assessment of model fit.
- Although sex differences in IR prevalence were reported, potential effect modification by sex or age on the association between TyG-based indices and IR was not formally examined. These analyses may provide additional clinical insight and should be addressed in future studies.

Finally, optimal cut-off values and diagnostic performance of TyG-related indices and the TG/HDL-C ratio may vary across ethnicities and regions; therefore, thresholds derived from the Turkish population should not be directly extrapolated to other populations without external validation.

### 4.2. Clinical and Public Health Implications

The TG/HDL-C ratio, the TyG index, and TyG-based indices are simple, reproducible, and low-cost measures. Because they rely on routinely obtained biochemical parameters, these markers have considerable potential for use in primary care and population-level screening.

In our study, the TyG index and TyG–WC demonstrated the greatest relevance after multivariable adjustment, with the TyG index emerging as the strongest independent predictor of IR. Their accessibility and affordability highlight the potential value of TyG-based indices in identifying individuals at increased metabolic risk and support their possible integration into future community-based screening strategies.

## 5. Conclusions

In conclusion, this large, province-wide, population-based study demonstrated that the TyG index and TyG–WC were independently associated with IR among Turkish adults, while the TG/HDL-C ratio also showed a weaker yet significant contribution. Although TyG–BMI and TyG–WC exhibited higher discriminatory performance in ROC analyses, the TyG index emerged as the most robust independent marker after multivariable adjustment. These findings support the potential utility of TyG-based indices for metabolic risk stratification in epidemiological and community-based contexts; however, their application in formal screening strategies should be considered exploratory and requires external validation and calibration in independent populations.

## Figures and Tables

**Table 1:** Baseline Demographic and Clinical Characteristics by Sex.

Variables	Women (*n* = 1045)	Men (*n* = 809)	Total (*n* = 1854)
Age (years)	45.9 ± 15.0	47.3 ± 15.3	46.5 ± 15.2
Height (cm)	156.6 ± 6.6	170.1 ± 7.1	162.4 ± 9.6
Weight (kg)	72.5 ± 14.5	79.0 ± 14.2	75.3 ± 14.7
Waist (cm)	92.4 ± 13.3	94.7 ± 12.1	93.4 ± 12.9
WHR	0.85 ± 0.08	0.92 ± 0.07	0.89 ± 0.08
BMI (kg/m^2^)	29.6 ± 6.0	27.3 ± 4.4	28.6 ± 5.5
IR (%)	29.7	23.9	27.2
DM (%)	15.4	11.6	13.8
HT (%)	41.1	32.0	37.2
Smoking status (%)			
Current user	8.6	35.8	20.5
Ex-smoker	5.4	30.5	16.3
No smoker	86	33.7	63.2
Education (%)			
Illiterate or literate, but no formal education	24.8	5.4	16.3
Formal education under high school	60.1	58.6	59.4
High school or above	15.1	36.0	24.2
Income status (%)			
<500 $	55.8	47.0	51.9
500–1000 $	28.9	32.4	30.4
>1000 $	15.3	20.6	17.6

Data are expressed as mean ± standard deviation (SD) for continuous variables and as percentages (%) for categorical variables.

**Table 2:** Baseline characteristics of participants according to IR status.

Variable	HOMA-IR < 2.46 Median (IQR) *n* = 1350	HOMA-IR ≥ 2.46 Median (IQR) *n* = 504	*p*-Value
Triglycerides (mg/dL)	98.9 (70.2–141.5)	144.4 (104.2–205.5)	<0.001
Fasting Glucose (mg/dL)	86.5 (78.7–94.2)	102.0 (90.5–125.2)	<0.001
HDL-Cholesterol (mg/dL)	51.0 (42.9–60.0)	45.2 (39.3–53.0)	<0.001
Height (cm)	162.0 (156.0–170.0)	160.0 (154.0–169.0)	0.003
Weight (kg)	72.0 (63.0–82.0)	82.0 (73.0–92.0)	<0.001
Waist Circumference (cm)	91.07 ± 12.53	99.52 ± 11.69	<0.001
Insulin (μU/mL)	6.32 (4.52–8.39)	15.10 (12.39–20.51)	<0.001
C-peptide (ng/mL)	1.86 (151–2.27)	3.21 (2.66–4.00)	<0.001

Data are expressed as median (IQR) or mean ± SD, as appropriate. Comparisons between groups were performed using the Mann–Whitney U test for continuous variables and the chi-square test for categorical variables.

**Table 3:** TyG-based indices and TG/HDL-C ratio according to IR status.

Variable	No IR (Mean ± SD)	With IR (Mean ± SD)	*p*-Value
TyG index	8.39 ± 0.56	8.91 ± 0.60	<0.001
TyG–BMI	231.54 ± 49.97	281.07 ± 50.63	<0.001
TyG–WC	766.56 ± 134.04	887.50 ± 125.18	<0.001
TyG–WHtR	4.73 ± 0.86	5.50 ± 0.82	<0.001
TG/HDL-C ratio	2.56 ± 2.18	3.79 ± 3.29	<0.001

**Table 4:** Correlations between HOMA-IR and TyG-based indices.

Variable	Pearson r	*p*-Value
TyG index	0.482	<0.001
TyG–BMI	0.515	<0.001
TyG–WC	0.484	<0.001
TyG–WHtR	0.480	<0.001
TG/HDL-C ratio	0.252	<0.001

**Table 5:** ROC analysis of TyG and TyG-based indices for predicting IR.

Variable	AUC	Cut-Off	Sensitivity (%)	Specificity (%)
TyG index	0.740	8.45	78.7	56.5
TyG–BMI	0.765	253.80	72.0	69.5
TyG–WC	0.751	864.27	72.0	69.3
TyG–WHtR	0.748	4.92	78.9	60.6
TG/HDL-C ratio	0.683	2.14	76.1	55.1

**Table 6:** Univariate logistic regression analysis for predictors of IR.

Variable	OR	95% CI (Lower–Upper)	*p*-Value
TyG index	4.68	3.81–5.75	<0.001
TyG–BMI	1.02	1.016–1.021	<0.001
TyG–WC	1.01	1.006–1.008	<0.001
TyG–WHtR	2.86	2.49–3.30	<0.001
TG/HDL-C ratio	1.21	1.16–1.26	<0.001

All TyG-based indices were significantly associated with IR in univariate analysis (*p* < 0.001).

**Table 7:** Multivariate Logistic Regression Models for IR.

Variable	OR	95% CI (Lower–Upper)	*p*-Value
TyG index	4.14	3.32–5.18	<0.001
TyG–BMI	0.96	0.94–0.97	<0.001
TyG–WC	1.00	1.00–1.01	0.032
TyG–WHtR	0.62	0.36–1.08	0.091
TG/HDL-C ratio	1.17	1.11–1.22	<0.001
Age	0.99	0.98–1.00	0.038
Sex (Male)	0.84	0.67–1.06	0.147
BMI (kg/m^2^)	1.14	1.11–1.16	<0.001

Each surrogate marker was evaluated in a separate model adjusted for age, sex, and BMI. Although statistically significant, TyG–BMI is collinear with BMI and cannot be interpreted as an independent predictor.

## Data Availability

The data presented in this study are available on reasonable request from the corresponding author. The data are not publicly available due to ethical and privacy restrictions.
